# Cellular and Molecular Mechanisms of Metformin Action

**DOI:** 10.1210/endrev/bnaa023

**Published:** 2020-09-08

**Authors:** Traci E LaMoia, Gerald I Shulman

**Affiliations:** 1 Department of Internal Medicine, Yale School of Medicine, New Haven, Connecticut; 2 Department of Cellular & Molecular Physiology, Yale School of Medicine, New Haven, Connecticut

**Keywords:** metformin, type 2 diabetes, hepatic gluconeogenesis, redox

## Abstract

Metformin is a first-line therapy for the treatment of type 2 diabetes, due to its robust glucose-lowering effects, well-established safety profile, and relatively low cost. While metformin has been shown to have pleotropic effects on glucose metabolism, there is a general consensus that the major glucose-lowering effect in patients with type 2 diabetes is mostly mediated through inhibition of hepatic gluconeogenesis. However, despite decades of research, the mechanism by which metformin inhibits this process is still highly debated. A key reason for these discrepant effects is likely due to the inconsistency in dosage of metformin across studies. Widely studied mechanisms of action, such as complex I inhibition leading to AMPK activation, have only been observed in the context of supra-pharmacological (>1 mM) metformin concentrations, which do not occur in the clinical setting. Thus, these mechanisms have been challenged in recent years and new mechanisms have been proposed. Based on the observation that metformin alters cellular redox balance, a redox-dependent mechanism of action has been described by several groups. Recent studies have shown that clinically relevant (50-100 μM) concentrations of metformin inhibit hepatic gluconeogenesis in a substrate-selective manner both in vitro and in vivo, supporting a redox-dependent mechanism of metformin action. Here, we review the current literature regarding metformin’s cellular and molecular mechanisms of action.

## Graphical Abstract

**Graphical Abstract:**
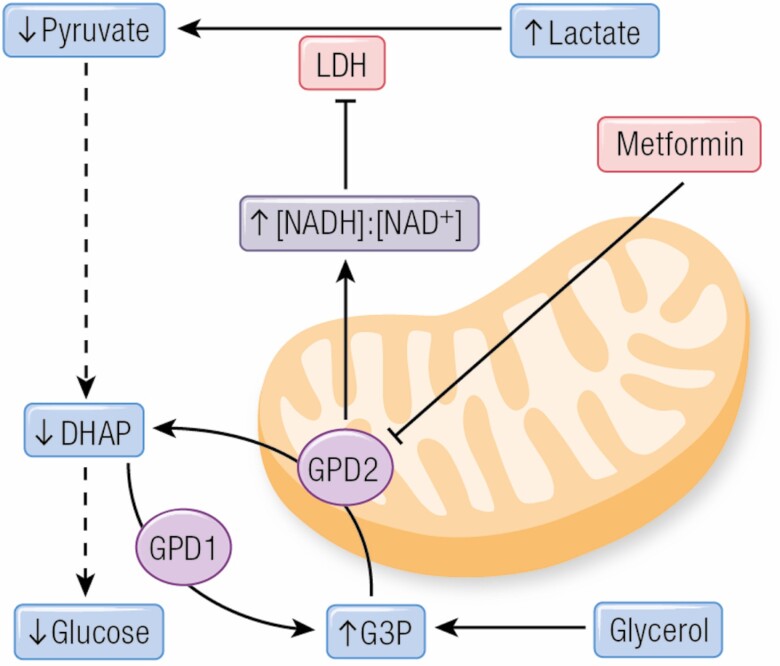

**ESSENTIAL POINTS**

- Metformin’s glucose-lowering effect in patients with poorly controlled type 2 diabetes is primarily due to inhibition of hepatic gluconeogenesis, while muscle and gut microbiota effects are largely secondary
- Hepatic gluconeogenesis is regulated through 4 distinct mechanisms: transcription, allosteric, substrate availability, and redox
- Metformin inhibition of complex I is only observed at millimolar concentrations that are not therapeutically relevant
- Metformin inhibits hepatic gluconeogenesis both in vitro and in vivo in a redox-dependent manner
- Increased cytosolic redox state, due to metformin inhibition of glycerol-3-phosphate dehydrogenase, is observed at clinically relevant concentrations, and this is the only proposed mechanism of action that predicts substrate-selective (glycerol and lactate) inhibition of hepatic gluconeogenesis

Metformin is the most widely prescribed drug for the treatment of type 2 diabetes (T2D) and is considered an “essential medicine” by the World Health Organization (1). Several studies have shown that metformin effectively improves glycemic control in patients with T2D, yet it rarely causes hypoglycemia due to its remarkable safety profile (2, 3). Metformin’s antidiabetic effect is primarily due to inhibition of hepatic gluconeogenesis; however, its mechanism of action remains a subject of debate. In this review, we will discuss proposed mechanisms of action of metformin in the context of initial studies in patients with T2D dating back several decades through the most recent studies to date.

We begin with a brief section (“Historical Overview”) summarizing the events that led from metformin’s initial discovery to its widespread clinical use. We then review early studies performed in patients with T2D (“Insights From Metformin’s Effects on Glucose Metabolism in Patients With Type 2 Diabetes”) that provided critical insights to metformin’s mechanism of action in humans. These initial studies provided key evidence showing inhibition of hepatic glucose production (HGP) as a major mechanism of metformin action and established a pattern of tissue distribution that led to the identification of the transporters required for metformin uptake by the liver. In the next section (“Regulation of Hepatic Gluconeogenesis”), we summarize the major mechanisms of hepatic gluconeogenesis regulation, including allosteric, transcriptional, substrate, and redox. Finally, we review the proposed mechanisms by which metformin inhibits hepatic gluconeogenesis (“Proposed Mechanisms by Which Metformin Inhibits Hepatic Gluconeogenesis”), focusing on metformin inhibition of complex I leading to reductions in hepatocellular energy charge and other downstream events (eg, adenosine monophosphate–activated protein kinase [AMPK] activation, fructose 1,6-bisphosphatase inhibition, inhibition of glucagon signaling), as well as an alternative hypothesis involving metformin induction of increased hepatocellular cytosolic redox state leading to decreased conversion of glycerol and lactate to glucose.

## Historical Overview

Metformin (1,1-dimethylbiguanide) originates from *Galega officinalis*, also known as the French lilac, a plant that has been used in folk medicine for centuries (4, 5). The first reference to the glucose-lowering effects in literature can be found in 1772, when it was described for the treatment of thirst and frequent urination (4). In the mid-19th century, chemical analysis of *G. officinalis* identified guanidine, a monoguanide compound which was later shown to have glucose-lowering effects in rabbits (6). Guanidine was quickly reported to have a poor toxicity profile, and derivatives were synthesized throughout the early 20th century, with the first reports of dimethylbiguanide synthesis in 1922 (6, 7). By the 1930s, however, interest in guanide-based compounds for the treatment of diabetes declined due to the rise in the use of insulin, which after its discovery by Banting and coworkers in 1921 was becoming widely available. It was not until 1957 that Jean Sterne ignited the reinvestigation of metformin by characterizing the pharmacodynamics of guanidine-based compounds in healthy and diabetic animals (8, 9).

Metformin was initially of little clinical interest due to its low potency, which demanded very high doses to effectively lower blood glucose in healthy animals (10). Instead, the United States and Europe began trials of phenformin and buformin, which ended early due to the emergence of severe lactic acidosis in many patients (11, 12). Consequently, the guanides garnered a poor reputation in the medical community and trials were widely discontinued. However, based on the earlier work of Jean Sterne, it was clear that hypoglycemia and lactic acidosis could likely be avoided with the less potent biguanide metformin, which had a better safety profile with fewer side effects (13).

Although there was widespread skepticism of metformin for the treatment of T2D due to the failure of phenformin, further studies in the 1980s and 1990s provided strong evidence for metformin’s glucose-lowering effects in patients with T2D with minimal side effects (14-19). Pagano et al reported that 4 to 6 weeks of metformin treatment caused a striking decrease in the insulin requirement of patients with type 1 diabetes (T1D) without inducing lactic acidosis (19), and subsequent studies showed that metformin effectively improved peripheral insulin sensitivity in both T1D and T2D patients (20, 21). Thus, the FDA ultimately approved metformin in 1994 and since then it has fundamentally altered our standard of care for patients with T2D.

By 1997, the results of the UK Prospective Diabetes Study (UKPDS) redefined our understanding of the long-term benefits of glycemic control in diabetic patients and the efficacy of various therapeutic interventions (2). In this seminal study, more than 5000 patients with recently diagnosed T2D were assigned to glucose-lowering treatment groups including dietary intervention, metformin, sulfonylurea, or insulin. The results clearly highlighted the beneficial outcomes of metformin, with patients randomized to this treatment group having reduced diabetes-related death and fewer hypoglycemic attacks than other pharmaceutical interventions (2). Metformin was subsequently recommended as a first-line therapy for the treatment of T2D.

In the 2 decades since the groundbreaking UKPDS, metformin has become the most widely prescribed drug for T2D worldwide, and was added to the World Health Organization’s list of essential medicines in 2011 (22). The intervening years have seen metformin research surge, as the mechanism of action of metformin has become a highly debated topic that has recently expanded beyond the diabetes field. The cancer field has begun to explore the antitumor effect of metformin as a potential treatment for certain cancers (23, 24), and the first trials of metformin for treating aging in healthy humans are currently underway (25, 26). Several mechanisms have been proposed linking metformin action and increased life- and health-span; most recently, metformin was shown to alleviate age-associated inflammation by increasing autophagy and improving mitochondrial function (27). Similarly, metformin has shown promise as a treatment for neurodegenerative diseases, including amyotrophic lateral sclerosis (ALS), by modulating dysregulated protein synthesis (28). Metformin has also been shown to improve cardiovascular outcomes in patients with and without T2D, which can be attributed to increased vascular function, improved lipid profiles, and potentially, as a side effect of metformin-induced weight loss (2, 29-33).

In summary, during the past century metformin has progressed from a recently synthesized compound of unknown therapeutic potential, to one of the most widely prescribed drugs for T2D worldwide. Yet, a consensus surrounding a mechanism of action remains elusive, and the benefit of metformin for chronic illnesses in nondiabetic patients has yet to be determined.

## Insights From Metformin’s Effects on Glucose Metabolism in Patients With Type 2 Diabetes

As a first-line therapy for the treatment of T2D, metformin is very effective at improving glycemic control in patients with T2D. However, metformin has also been shown to have paradoxical effects on hepatic glucose metabolism in nondiabetic patients (34, 35). Thus, early clinical studies in T2D patients investigating metformin’s mechanism of action have provided important insights into our current understanding of its glucose-lowering effects (3, 18, 36, 37).

Insulin resistance is a key feature of T2D, leading to impaired insulin-stimulated glucose uptake and decreased insulin suppression of HGP (16, 38). However, in patients with poorly controlled T2D, increased gluconeogenesis is the major factor contributing to increased rates of HGP and fasting hyperglycemia (39). Traditionally, metformin is thought to primarily act on the liver, and clinical studies in patients with T2D have confirmed that the primary mechanism of action is inhibition of HGP without concomitant increases in plasma insulin concentrations (3, 36, 37, 40-42). Metformin has also been shown to have effects on peripheral glucose metabolism (3, 43) and intestinal glucose metabolism (44, 45), which are reviewed in the sections below.

### Pharmacokinetics

Metformin has an oral bioavailability of 50% to 60% and, following intestinal absorption, enters the portal vein and accumulates in the liver. Patients prescribed metformin receive doses of 1 g/day to 2 g/day [or ~20 mg/(kg-day)], leading to plasma metformin concentrations of ~10 µM to ~40 µM (46-49). However, as previously discussed by Madiraju et al (48, 50), “therapeutic” metformin concentrations are notably discordant in the literature, especially between in vitro (Table 1) and in vivo (Table 2) studies. Previous in vitro and animal studies have reported “therapeutic” plasma metformin concentrations ranged from ~1 µM to ~700 µM, and many of the studies failed to cite a supporting reference (49). However there appears to be a general consensus in the literature that plasma concentrations of metformin in humans who are being treated with metformin range between 0.1 mg/L and 4 mg/L (~1 µM to ~40 µM) (Table 2).

**Table 1.** Metformin Concentrations Used in In Vitro Studies

In Vitro		
Study	Model	Metformin Concentration in Media
El-Mir et al., 2000	Hepatocytes	10 mM
Owen et al., 2000	Isolated mitochondria	10 mM
Zhou et al., 2001	Hepatocytes	0.02-2 mM
Hawley et al., 2002	H4IIE cells	0.05-5 mM
Foretz et al., 2010	Hepatocytes	0.25-2 mM
Palenickova et al., 2011	Isolated mitochondria	1.25-20 mM
Logie et al., 2012	H4IIE cells	0.5-5 mM
Fullerton et al., 2013	Hepatocytes	0.5 mM
Miller et al., 2013	Hepatocytes	0.01-1 mM
Wheaton et al., 2014	HCT 116 cells	0.25-4 mM
Cao et al., 2014	Hepatocytes	0.02-1 mM
Madiraju et al., 2014	Hepatocytes and purified enzyme	0.05-0.25 mM
Guo et al., 2017	MCF-7 cells	0.75-5 mM
Madiraju et al., 2018	Hepatocytes	0.01 mM
Cameron et al., 2018	Hepatocytes and H4IIE cells	.025-10.9 mM
Li X et al., 2019	S. cerevisiae	0.1-100 mM
Alshawi et al., 2019	Hepatocytes	0.05-5 mM
Li W et al., 2019	Isolated mitochondria & astrocytes	0.02-2 mM
Xie et al., 2020	15 cancer cell lines	6-200 mM

In vitro studies discussed in this review are included here with the model and concentration of metformin in the media listed for each study. Many of these in vitro studies use supra-pharmacological (>1 mM) metformin concentrations.

**Table 2.** Metformin Doses Used in In Vivo Studies

Study	Model	Dose	Route of Administration	Duration/Frequency	Peak/Observed Plasma Metformin	Decreased Plasma Glucose?
Zhou et al., 2001	Rat	~300 mg/kg	Oral	5 days	NR	No
Shaw et al., 2005	High-fat-fed mice	250 mg/kg/d	I.P. injection	3 days	NR	Yes
Foretz et al., 2010	Mouse	50-300 mg/kg	Oral	Acute	NR	Yes
Foretz et al., 2010	Mouse	20-50 mg/kg/d	Oral	5 days	NR	NR
Fullerton et al., 2013	Mouse	150-400 mg/kg	I.P. injection	Acute	NR	NR
Fullerton et al., 2013	High-fat-fed mice	50 mg/kg/d	I.P. injection	6 weeks	NR	Yes
Miller et al., 2013	Mouse	250-500 mg/kg	Oral	Acute	NR	Yes
Wheaton et al., 2014	Mouse	1.25 mg/mL	Oral	Ad libitum drinking water, 26 days	NR	No
Cao et al., 2014	High-fat-fed mice	50 mg/kg/d	Oral	Ad libitum drinking water, 6 weeks	NR	NR
Madiraju et al., 2014	Rat	50 mg/kg	Intravenous	Acute	74 µM (0.5 hr)	Yes
Madiraju et al., 2014	Rat	100 mg/kg	Intravenous	Acute	345 µM (0.5 hr)	NR
Madiraju et al., 2014	Rat	250 mg/kg	Intravenous	Acute	1300 µM (0.5 hr)	NR
Madiraju et al., 2014	Rat	50 mg/kg/d	I.P. injection	30 days	NR	Yes
Lewis et al., 2016	Rat	~2.8 mg/mL	Oral	Ad libitum drinking water, 4 weeks	NR	NR
Madiraju et al., 2018	Rat	50 mg/kg	Intraportal	Acute	~130 µM (0.5 hr)	Yes
Madiraju et al., 2018	Rat	3.5 mg/mL	Oral	Ad libitum drinking water, 14 days	~15 µM*	Yes
Qi et al., 2018	Rat	50 mg/kg	Intravenous	Acute	NR	NR
Hunter et al., 2018	Mouse	250 mg/kg	Oral	Acute	~150 µM (1 hr)*	Yes
Hunter et al., 2018	Mouse	1.875 mg/[kg-min]	Intravenous	120-minute infusion	~175 µM (end of infusion)*	N/A
Hunter et al., 2018	Mouse	3.75 mg/[kg-min]	Intravenous	120-minute infusion	~350 µM (end of infusion)*	N/A
Lalau et al., 1995	Human	1.7-2.55 g/day	Oral	“Long-term”	~4 µM*	NR
Timmins et al., 2005	Human	1 g twice/day	Oral	5 weeks	~10 µM (~6 hr)	NR
Frid et al., 2010	Human	1-3 g/day	Oral	2 months	0.1-20 µM*	NR
Madiraju et al., 2018	Human	1 g	Oral	Acute	~20 µM (~3 hr)	NR
Madiraju et al., 2018	Human	1 g twice/day	Oral	Chronic	35 µM (~3 hr)	NR

In vivo rodent and human studies discussed in this review are included here with the metformin dose, route of administration, duration, and frequency of treatment listed for each study. Peak plasma and liver metformin concentrations are also listed if they are reported in the original study, with the time of measurement listed in parentheses. *Time course not available, plasma metformin concentrations displayed as single time point. Abbreviations: NR, not reported.

In nondiabetic patients treated with 1g metformin orally, plasma concentrations reach 25 µM within 3 hours of administration, while diabetic patients chronically administered 1g metformin twice daily reach peak plasma metformin concentrations of ~40 µM (48, 50). Similar values are reported in healthy volunteers treated with either 1g of the instant or extended-release formulations of metformin for 1 week, with peak plasma concentrations ranging from 5 µM to 10 µM (47). Another study in patients with T2D given 1 g/day to 3 g/day for 8 weeks reported median plasma metformin concentrations of ~10 µM (51). Taken together, although additional pharmacokinetic studies would be beneficial to the field, it is likely that the true range of therapeutic plasma metformin concentrations in humans is ~10 µM to ~40 µM. However, as discussed below, hepatic concentrations of metformin in humans are probably 2- to 3-fold higher than this due to portal vein absorption and first pass uptake of metformin by the liver following oral dosing.

Based on the reported plasma metformin concentrations in patients with T2D orally administered metformin, several rodent studies have been conducted to determine clinically relevant doses and routes of administration. Acute intravenous metformin administration of 50, 100, and 250 mg/kg resulted in peak plasma concentrations of 74, 345, and 1300 µM, respectively, and liver metformin concentrations reached ~100 µM following intravenous 50 mg/kg metformin administration in awake rats (50). Oral administration of metformin in rodents with *ad libitum* access to metformin-treated drinking water results in inconsistent plasma metformin concentrations, possibly due to variability in drinking and/or absorption from the gut, with studies reporting plasma concentrations ranging from 5 µM to 180 µM (48, 52, 53). As noted above, more relevant than systemic plasma concentrations following oral metformin dosing are plasma concentrations of metformin in the portal vein (~50-60 µM) following oral metformin dosing (50 mg/kg), which similar to other orally ingested substances range 2- to 3-fold higher than systemic metformin concentrations (~10-40 µM) (54, 55). In regard to hepatic metformin concentrations following oral dosing, Wilcock and Bailey reported liver metformin concentrations of ~180 μM in mice following 50 mg/kg orally administered metformin, which is consistent with the values reported by Madiraju et al (100 μM) following acute intravenous 50 mg/kg metformin treatment in awake rats (48). Assessing liver metformin concentrations in humans is challenging, due to the necessity of obtaining liver tissue for this measurement; however, ^11^C-metformin has recently been used to noninvasively assess metformin biodistribution. Using this approach, Gormsen et al found significant liver ^11^C-metformin uptake following oral administration of the ^11^C-metformin tracer. They reported a tissue-to-blood ratio double what was observed after intravenous administration, which is consistent with the higher hepatic to systemic plasma metformin concentrations observed in rodent studies following oral metformin administration (56).

Investigation into the mechanism of action of metformin has yielded conflicting results, which may be due to the variability in doses and route of administration. Many, if not most, in vitro (Table 1) and in vivo (Table 2) studies that have examined the cellular and molecular mechanisms by which metformin reduces HGP have utilized supra-pharmacological (>1 mM) metformin doses that may not be clinically relevant (57-61). Furthermore, few in vivo studies report plasma and tissue metformin concentrations (Table 2). This should be a common practice, as pharmacokinetic properties differ between species, as well as treatment regimens. One of the most widely studied mechanisms of metformin action, complex I inhibition leading to altered hepatic energy charge, is only observed at millimolar concentrations. Approximately 2 mM metformin is required to alter adenine nucleotides, and significant complex I inhibition has consistently been reported to occur only with metformin concentrations of at least 1 mM to 5 mM (57-59, 62, 63). Thus, to effectively assess clinically relevant mechanisms of metformin action, metformin dosage, route of administration, and model system should be taken into consideration.

Defining clinically relevant doses of metformin to be used in rodent studies is difficult, given that portal vein and liver concentrations of metformin following oral ingestion of metformin in humans are unknown. However, based on measured plasma concentrations of metformin in humans following oral ingestion of 1 g of metformin ranging from 20 to 30 μM and assuming roughly 3-fold higher gradient of metformin in the portal vein relative to systemic concentrations following oral ingestion, then hepatic exposure to metformin can be roughly estimated to be 60 to 90 μM. Based on these estimates Madiraju et al have shown that oral dosing of metformin (50-100 mg/kg) in rats achieves comparable hepatic exposure (~50-100 μM) to humans taking 1 g of metformin twice daily (Table 2). Whereas doses of metformin ≥250 mg/kg will expose the liver to hepatic metformin concentrations of >1 mM (Table 2), which are supra-pharmacologic and will likely result in non–clinically relevant effects of metformin (eg, complex I inhibition) on hepatic glucose metabolism.

### Liver specificity

Metformin exists in a positively charged state under physiologic conditions, suggesting that a transporter is likely necessary for metformin to cross plasma membranes. Wang et al reported that metformin uptake requires organic cation transporter 1 (OCT1), which is highly expressed in the liver, kidney, and intestine (64). It was later shown that OCT3 and MATE1 transporters also play a role in metformin uptake (46, 64, 65). The tissue distribution of metformin is consistent with expression of these transporters; in humans, labeled metformin accumulates to much higher concentrations in the liver, kidney, and small intestine, with relatively little uptake peripherally (56). Polymorphisms of OCT1 are also shown to alter the pharmacokinetics of metformin and reduce therapeutic action; however, whether genetic variation plays a role in the variable clinical response to metformin is still unclear (66-68).

The first clinical indication that metformin directly affects HGP, as opposed to augmenting insulin secretion or increasing glucose disposal, was borne out by clinical studies in the 1990s. Hyperinsulinemic-euglycemic clamp studies performed on patients with T2D before and after chronic metformin treatment consistently showed that metformin decreased rates of HGP, while insulin-stimulated peripheral glucose uptake was increased in some studies and unchanged in others (3, 37, 40, 41). Further investigation, utilizing ^13^C magnetic resonance spectroscopy to directly measure rates of net hepatic glycogenolysis and gluconeogenesis in combination with stable isotopes to measure rates of endogenous glucose production, established that metformin-induced reductions in rates of HGP could be entirely attributed to decreased rates of hepatic gluconeogenesis, rather than reductions in rates of net hepatic glycogenolysis (42). This was confirmed independently in these same subjects using ^2^H_2_O to quantify rates of gluconeogenesis (42). Additionally, these studies showed that the antidiabetic effect of metformin can be primarily attributed to reductions in hepatic glucose metabolism, rather than increased insulin secretion and improved pancreatic β-cell function, as plasma insulin concentrations were either reduced or unchanged in these studies. Consistent with these results, Inzucchi et al (36) demonstrated that metformin treatment resulted in an ~20% reduction in HGP in patients with poorly controlled T2D. These results were confirmed in a placebo-controlled study by Cusi et al, who demonstrated that metformin treatment resulted in an ~15% reduction in HGP in patients with poorly controlled T2D (69). Contrary to these studies, Gormsen et al reported a paradoxical increase in HGP in nondiabetic patients and patients with recent-onset T2D (70). However, this may be due to a pronounced compensatory increase in plasma glucagon that was observed in these nondiabetic subjects (71).

Taken together, evidence in support of a liver-specific mechanism of metformin action includes clinical data showing pronounced inhibition of HGP in patients with T2D given metformin in the absence of major changes in peripheral glucose disposal (as discussed below) and/or insulin secretion. Furthermore, the high portal vein concentrations of metformin in addition to the well-established pattern of metformin accumulation in the liver, kidney, and intestine due to the expression of cation transporters is consistent with these findings. Thus, it is likely that metformin’s beneficial therapeutic effects can mostly be attributed to reductions in rates of hepatic gluconeogenesis resulting in decreased rates of HGP.

### Muscle effects

In addition to inhibiting hepatic gluconeogenesis and HGP, metformin has also been shown to act on skeletal muscle to increase insulin-stimulated glucose uptake (35, 40, 43). An early study by DeFronzo et al found that metformin increased whole-body insulin-stimulated tissue glucose uptake in patients with T2D, but this effect was exclusive to obese patients with T2D and could not be entirely attributed to skeletal muscle glucose uptake (3). Additionally, AMPK activity and phosphorylation was increased in muscle biopsies from patients with T2D following metformin treatment (72). Consistent with these results, Inzucchi et al found that 3 months of metformin treatment (1000 mg twice a day) increased insulin-stimulated peripheral glucose uptake by 13% along with a ~20% reduction in rates of HGP, resulting in a 58 mg/dL reduction in fasting plasma glucose concentration in patients with poorly controlled T2D (36). However, it is likely that this effect of metformin to promote increased insulin-stimulated peripheral glucose uptake is an indirect effect related to reductions in glucose toxicity (73). In support of this hypothesis, Yu et al observed no effects of metformin (850 mg twice/day) treatment on insulin-stimulated peripheral glucose metabolism in T2D patients rendered normoglycemic following 4 weeks of continuous subcutaneous insulin (74). Later studies using positron emission tomography (PET) imaging demonstrated that the observed increase in whole-body glucose uptake could be dissociated from skeletal muscle-specific effects (75).

Taken together, these studies indicate that metformin’s effect to increase insulin-stimulated peripheral glucose uptake is secondary to improved glycemic control and reversal of glucose toxicity, which can mostly be attributed to metformin’s ability to directly inhibit hepatic gluconeogenesis and HGP.

### Intestinal effects

Metformin has an oral availability of about 60% and is shown to accumulate in the small intestine, as well as the liver and kidney, due to the expression of the OCT1, OCT3, and PMAT transporters in these tissues (46, 64). Thus, several studies have described an intestinal mechanism for metformin’s glucose-lowering effects and the gastrointestinal side effects that are observed in some patients. Importantly, observational studies have reported intestinal side effects in as low as 16% and as high as 62% of patients, leading to metformin intolerance in ~5% of patients (76-78)

In recent years, the clinical benefits of metformin have been linked to alterations in gut microbiome composition, intestinal glucose uptake, and hormone (eg, growth differentiation factor 15 [GDF15], glucagon-like peptide-1 [GLP-1]) secretion (44, 45, 79-82). Several groups have reported significant metformin-induced shifts in microbiome composition in T2D patients treated with metformin compared with placebo; however, it is unclear whether these changes to the gut microbiota are responsible for the glucose-lowering effects of metformin or secondary in nature. In support of a causal role for metformin-induced changes being responsible for metformin’s glucose-lowering effects, germ-free mice were given a fecal transplant from patients before or after metformin treatment, which improved glucose tolerance in mice receiving the post-metformin fecal transplant (45). However, these studies need to be replicated in humans to establish the clinical relevance of this mechanism.

Recently, intestinal metformin action has been linked to the weight loss and reduced appetite that is frequently observed in patients (82-84). Elevated serum GDF15 has been observed in patients with T2D, and this was recently linked to metformin treatment (85). This emerging story describes a potential mechanism in which metformin-induced activation of the integrated stress response pathway leads to GDF15 secretion, which improves glycemic regulation and reduces appetite (82, 84). Interestingly, metformin stimulates GDF15 expression and secretion from hepatocytes in vitro; however, Coll et al report significantly increased intestinal GDF15 expression without changes in hepatic GDF15 expression in mice treated with metformin orally (82). Thus, whether the liver is also involved with metformin-induced secretion of GDF15 requires further investigation.

Additional mechanisms implicating intestinal metformin action include augmented GLP-1 secretion, delayed gastric emptying, and altered enterocyte glucose metabolism (86). Studies investigating the effect of metformin on GLP-1 secretion have yielded conflicting results, with some groups reporting a direct effect on GLP-1 expression, indirect effects through dipeptidyl peptidase-4 (DPP4) activity, or no effect on GLP-1 at all (87, 88). The effect of metformin on intestinal glucose uptake is well-established through PET-computed tomography studies, and metformin treatment is often discontinued prior to PET imaging to avoid confounding results (86). However, the therapeutic relevance of this mechanism is unclear

## Regulation of Hepatic Gluconeogenesis

Hepatic glucose production (HGP) reflects the net contributions of hepatic gluconeogenesis, glycogenolysis, glycogen synthesis, and glycolysis. That approximately 85% to 90% of endogenous glucose production following an overnight fast is attributed to HGP highlights the importance of HGP to whole-body glucose metabolism and glycemic control (89). Furthermore, in the overnight fasted state, about 50% of HGP can be attributed to hepatic gluconeogenesis and, in patients with poorly controlled T2D, an increased rate of hepatic gluconeogenesis is the primary driver of fasting hyperglycemia (39, 90).

**Figure 1.**
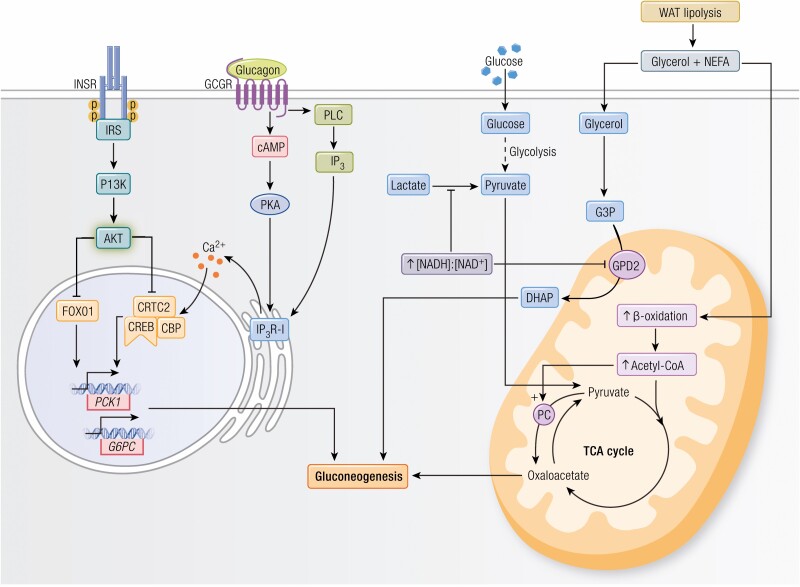
Regulation of hepatic gluconeogenesis. Hepatic gluconeogenesis is regulated through allostery, substrate availability, redox balance, and gene expression. WAT lipolysis produces glycerol and NEFA, which can both independently stimulate hepatic gluconeogenesis. NEFA enters mitochondrial β-oxidation which produces acetyl-CoA, an allosteric activator of PC. PC catalyzes the conversion of pyruvate to oxaloacetate, which can directly enter the gluconeogenic pathway. On the other hand, glycerol is itself a gluconeogenic substrate. Glycerol from WAT lipolysis is phosphorylated to G3P and converted to DHAP, a gluconeogenic intermediate, by GPD2. The reaction catalyzed by GPD2 is dependent on cellular redox state and is inhibited by a high [NADH]:[NAD^+^] ratio. Similarly, gluconeogenesis from lactate is redox-regulated, because the conversion of lactate to pyruvate by LDH is inhibited by a high [NADH]:[NAD^+^] ratio. Transcriptional regulation of *PCK1* and *G6PC* is coordinated by the opposing actions of glucagon and insulin. When insulin binds its receptor, AKT is activated and promotes nuclear exclusion of FOXO1, which decreases gluconeogenic gene expression. In contrast, glucagon binds the glucagon receptor and promotes IP_3_R-I-mediated ER Ca^++^ release. This in turn activates CRTC2, which forms a complex with CREB and CBP, and promotes transcriptional upregulation of *PCK1* and *G6PC*. Abbreviations: CBP, CREB-binding protein; CREB, cAMP-responsive element-binding protein 1; CRTC2, CREB-regulated transcription co-activator 2; DHAP, dihydroxyacetone phosphate; DKI, double knock-in; FOXO, Forkhead box O; G3P, glycerol-3-phosphate; GCGR, glucagon receptor; INSR, insulin receptor; LDH, lactate dehydrogenase; NEFA, nonesterified fatty acid; PC, pyruvate carboxylase; WAT, white adipose tissue;

As discussed above, metformin’s glucose-lowering effect is primarily due to suppression of hepatic gluconeogenesis. Although the precise mechanism of metformin action on gluconeogenesis remains a subject of debate, a fundamental understanding of the underlying mechanisms of gluconeogenic regulation is necessary to critically evaluate the existing hypotheses of metformin action. In this section, we consider the following mechanisms of gluconeogenic regulation: transcriptional, allosteric, substrate, and redox. We also discuss proposed mechanisms of metformin action related to the regulation of hepatic gluconeogenesis through each of these processes (Fig. 1).

### Transcriptional

Alterations in expression of gluconeogenic genes under the control of Forkhead box O (FOXO) and cAMP-responsive element-binding protein 1 (CREB) are often used as a readout of gluconeogenic regulation. The CREB–CREB-binding protein (CBP)–CREB-regulated transcription co-activator 2 (CRTC2) transcriptional complex increases expression of glucose-6-phosphatase (*G6pc*) and phosphoenolpyruvate carboxykinase 1 (*Pck1*), 2 gluconeogenic genes (91, 92). The formation of this complex is stimulated by glucagon and catecholamines, as well as by fasting conditions. Additionally, the FOXO family of transcription factors stimulate *G6pc* and *Pck1* expression. In response to insulin activation of AKT, FOXO proteins are phosphorylated and excluded from the nucleus, thus negatively regulating gluconeogenic gene expression (91).

The physiologic relevance of acute transcriptional regulation, however, is challenged by studies showing that genetic deletion of hepatic signaling mediators, such as INSR, AKT, and FOXO1, do not cause defects in insulin suppression of HGP during a hyperinsulinemic-euglycemic clamp (93, 94), suggesting extrahepatic mechanisms for gluconeogenic regulation. Although *G6pc* and *Pck1* expression were traditionally considered regulatory nodes of gluconeogenesis, protein expression of these enzymes has in fact been dissociated from gluconeogenic flux in vivo (94-97). Furthermore, there is little evidence to support a role for transcriptional dysregulation in T2D. Both rodents and humans with T2D show unaltered gluconeogenic gene and protein expression, further dissociating transcriptional control of gluconeogenesis from hyperglycemia (96).

Metformin has been proposed to transcriptionally regulate hepatic gluconeogenesis through several mechanisms. First, it has been suggested that metformin antagonizes hepatic glucagon signaling by decreasing cyclic AMP accumulation, thus preventing CREB-mediated transcription of gluconeogenic genes (98, 99). However, the clinical relevance of this mechanism was challenged by a study of prediabetic individuals demonstrating that metformin does not reduce glucagon-stimulated HGP (71). Additionally, metformin has been proposed to activate AMPK, leading to downregulation of gluconeogenic gene expression. The mechanistic link between metformin and AMPK activation will be discussed extensively in a later section (“Proposed Mechanisms by Which Metformin Inhibits Hepatic Gluconeogenesis”); however, AMPK activation is suggested to mediate disassembly of the CREB transcriptional complex, leading to reduced *G6pc* and *Pck1* expression (62). This mechanism is questioned by data showing that metformin inhibits HGP in the absence of transcriptional changes, and even in a mouse model overexpressing G6PC and PCK1 (59). The discrepancy between these studies may be a product of the supra-pharmacological metformin concentrations (10 mM) utilized in the study by He et al (62).

Moreover, several groups have reported rapid inhibition of gluconeogenesis following acute treatment with guanides or biguanides, which would not be achievable with transcriptional regulation (50, 59). Thus, while transcriptional regulation may determine maximal gluconeogenic capacity, it cannot explain acute reductions in gluconeogenic flux induced by guanides/biguanides treatment.

### Allosteric (acetyl-coenzyme A)

Hepatic gluconeogenesis is regulated by acetyl-coenzyme A (acetyl-CoA), an allosteric activator of pyruvate carboxylase (94, 100-102). Pyruvate carboxylase catalyzes the conversion of pyruvate to oxaloacetate, a key anaplerotic reaction that supplies carbon for gluconeogenesis; it is also the first committed step in the gluconeogenic pathway (103).

Following white adipose tissue (WAT) lipolysis, nonesterified fatty acids (NEFA) from the adipocyte are taken up by the liver, where β-oxidation produces acetyl-CoA, which subsequently binds to and allosterically activates pyruvate carboxylase (94). This extrahepatic mechanism of liver gluconeogenic regulation plays an important role in the maintenance of euglycemia, as hepatic insulin signaling is not sufficient to suppress hepatic gluconeogenesis. Studies in mice have shown that deletion of the hepatic insulin receptor in vivo is insufficient to prevent insulin suppression of HGP (94, 104). Similarly, as described in the preceding section, mice lacking hepatic insulin signaling molecules (eg, INSR, AKT) have normal hepatic insulin action (93, 94).

These findings are consistent with an indirect mechanism for the regulation of hepatic gluconeogenesis (105). Although there was evidence for pyruvate carboxylase regulation by acetyl-CoA as early as the 1960s, further investigation of the physiological relevance of this mechanism was limited by the inability to measure acetyl-CoA in vivo due to its rapid degradation (102). This challenge was addressed with the development of a novel liquid chromatography–tandem mass spectrometry (LC-MS/MS) technique that demonstrated that not only is hepatic acetyl-CoA content decreased by insulin, but inhibition of insulin suppression of WAT lipolysis is sufficient to prevent insulin suppression of hepatic gluconeogenesis (94). Taken together, dysregulated WAT lipolysis likely promotes increased rates of hepatic gluconeogenesis in patients with poorly controlled T2D indirectly through this allosteric mechanism. Indeed, individuals with T2D have elevated plasma NEFA, potentially indicating impaired lipolytic control (106-108). However, further studies are needed to clarify this association.

### Substrate (glycerol)

Hepatic gluconeogenesis is also indirectly regulated by glycerol delivery to the liver by WAT lipolysis, which contributes about 20% to 30% of hepatic gluconeogenesis (94, 100, 105). In contrast to allosteric control of hepatic gluconeogenesis by NEFA-derived acetyl-CoA, glycerol from WAT lipolysis increases gluconeogenesis and HGP by a substrate-push mechanism (109). Glycerol enters the gluconeogenic pathway when it is phosphorylated and converted to dihydroxyacetone phosphate (DHAP) by mitochondrial glycerol-3-phosphate dehydrogenase (GPD2). The reaction catalyzed by GPD2 is also redox-dependent and inhibited by an increase in the cytosolic redox state, which will be discussed in detail below in “Redox.”

The importance of the regulation of HGP by peripheral mechanisms became more widely recognized in the 1990s when it was discovered that peripheral, rather than portal, insulin concentrations are highly correlated with HGP suppression (110-112). Two studies showed that portal infusions of insulin in dogs reduced HGP; however, suppression of HGP tracked very closely with peripheral insulin levels rather than portal insulin (112, 113). Further evidence for indirect regulation of gluconeogenesis came from studies reporting increased glycerol turnover and gluconeogenesis from glycerol in type 2 diabetic patients compared with healthy controls (114, 115). Additionally, infusion of acetate and glycerol to maintain intrahepatic acetyl-CoA concentration and glycerol turnover respectively in awake rodents is sufficient to prevent insulin suppression of hepatic gluconeogenesis, indicating that regulation of WAT lipolysis by insulin indirectly regulates hepatic gluconeogenesis and maintains euglycemia (94).

One proposed mechanism of action for metformin is inhibition of GPD2, leading to reduced hepatic gluconeogenesis in a substrate-specific, redox-dependent manner. This is supported by the observation that acute metformin treatment increases plasma glycerol and hepatic glycerol-3-phosphate (G3P) concentrations in rodents, indicating reduced gluconeogenesis from glycerol (48, 50). At clinically relevant concentrations, metformin is shown to inhibit GPD2, leading to increased cytosolic redox state and decreasing gluconeogenesis from redox-dependent substrates. This proposed mechanism of action will be described at length in “Proposed Mechanisms by Which Metformin Inhibits Hepatic Gluconeogenesis.”

### Redox

Redox regulation of hepatic gluconeogenesis is dependent on both the [NADH]:[NAD^+^] ratio and the nature of the gluconeogenic substrate (116, 117). Redox balance is maintained by the continuous function of 2 redox shuttles: the malate-aspartate shuttle and the α-glycerophosphate shuttle. Perturbation of this balance of reducing equivalents can directly impact gluconeogenesis from redox-dependent substrates. Lactate, which reduces NAD^+^ to NADH during its conversion to pyruvate by lactate dehydrogenase, and glycerol, which feeds into the α-glycerophosphate redox shuttle through GPD2, are considered redox-dependent substrates. Conversely, alanine, pyruvate, and DHAP are redox-independent because their entry to the gluconeogenic pathway does not require NAD^+^ or NADH. Simply put, this means that a reduced cytosol, with a high [NADH]:[NAD^+^] ratio, will inhibit gluconeogenesis from lactate and glycerol, but not pyruvate, alanine, and DHAP.

This regulatory mechanism is especially pertinent in the context of obesity and T2D due to dysregulated WAT lipolysis and increased glycerol supply to the liver (114, 115, 118). Therefore, inhibition of gluconeogenesis from glycerol may disproportionately benefit individuals with poorly controlled T2D with dysregulated WAT lipolysis.

Metformin inhibition of GPD2 has been shown to increase cytosolic redox by disrupting the α-glycerophosphate redox shuttle, leading to an increase in the cytosolic redox state (increased cytosolic [NADH]:[NAD^+^]) resulting in inhibition of gluconeogenesis specifically from glycerol and lactate (48, 50). Therefore, in support of the GPD2/redox hypothesis of metformin action, the paradoxical effects of metformin in obese versus lean patients may be related to the differing contribution of glycerol to gluconeogenesis in these patients. Importantly, this substrate-specific effect that is observed with metformin treatment is not predicted by any other proposed mechanisms of metformin action (48, 50). Furthermore, this substrate-specific effect of metformin to inhibit gluconeogenesis would also explain why hypoglycemia is rarely observed in patients taking metformin given that other substrates (eg, alanine and other amino acids) are still able to contribute to gluconeogenesis. This proposed mechanism of action will be discussed further below.

## Proposed Mechanisms by Which Metformin Inhibits Hepatic Gluconeogenesis

### Complex I inhibition

**Figure 2.**
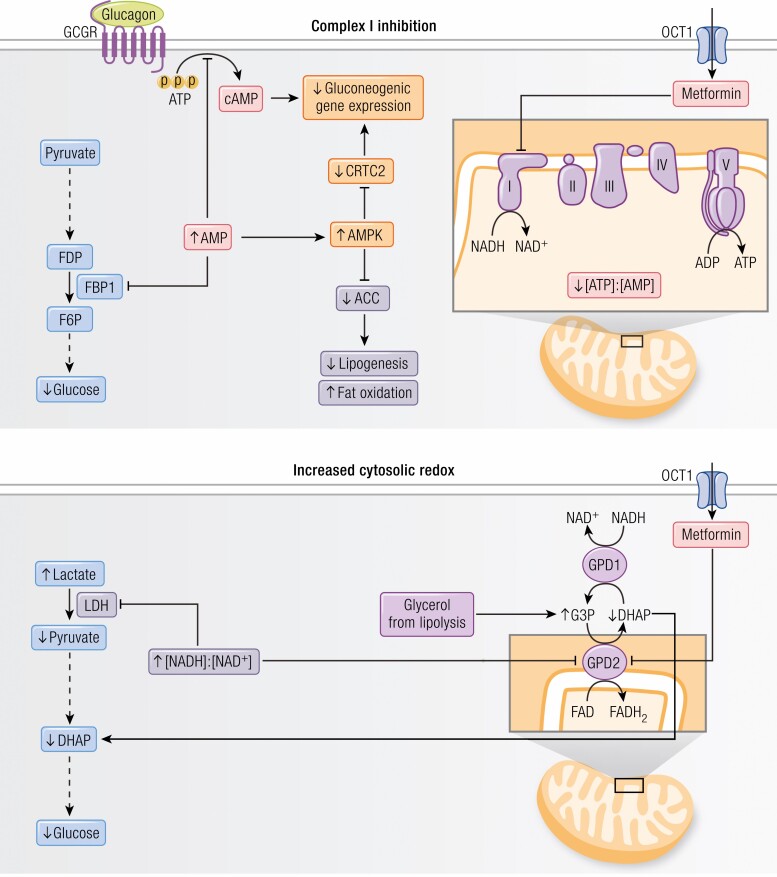
Proposed mechanisms of metformin action. Complex I inhibition (top panel). Inhibition of complex I is central to several proposed mechanisms of metformin action. Following complex I inhibition, AMPK is activated by increased AMP levels, leading to inhibition of CRTC2 and preventing formation of the CREB-CBP-CRTC2 complex (orange boxes). AMPK also phosphorylates and inhibits ACC1 and 2, which promotes fat oxidation and decreases lipogenesis (purple boxes). Additionally, increased AMP is proposed to inhibit hepatic gluconeogenesis independently of AMPK. High AMP prevents glucagon-stimulated production of cAMP and therefore antagonizes hepatic glucagon action (pink boxes). AMP also allosterically inhibits FBP1, which directly inhibits the gluconeogenic pathway (blue boxes). Increased cytosolic redox (bottom panel). Metformin inhibition of GPD2 reduces the conversion of G3P to DHAP which impairs gluconeogenesis from glycerol and simultaneously increases the cytosolic [NADH]:[NAD^+^] ratio. LDH is inhibited by the increased [NADH]:[NAD^+^] ratio, thus reducing gluconeogenesis from lactate. Metformin inhibition of GPD2 is the only proposed mechanism that is independent of Complex I inhibition and produces substrate-selective inhibition of gluconeogenesis. Abbreviations: ACC, acetyl-CoA carboxylase; AMPK, adenosine monophosphate–activated protein kinase; CBP, CREB-binding protein; CREB, cAMP-responsive element-binding protein 1; CRTC2, CREB-regulated transcription co-activator 2; DHAP, dihydroxyacetone phosphate; FBP, fructose 1,6-bisphosphatase; FDP, fructose 1,6-diphosphate; G3P, glycerol-3-phosphate; GLP-1, glucagon-like peptide-1; GPD, glycerol-3-phosphate dehydrogenase; LDH, lactate dehydrogenase; OCT1, organic cation transporter 1.

Inhibition of complex I activity is probably the most widely studied and commonly implicated mechanism of metformin’s glucose-lowering effect (Fig. 2, top panel). This hypothesis emerged in the early 2000s when 2 groups reported robust inhibition of complex I following metformin treatment in vitro (57, 58). These data are consistent with previous studies performed more than 50 years ago demonstrating that phenformin and other guanides inhibit complex I activity (119-121). Complex I is the site of NADH contribution to the mitochondrial proton gradient and, given the energetic cost of gluconeogenesis, its inhibition was linked to decreased HGP. Several mechanisms by which complex I inhibition leads to suppression of gluconeogenesis are proposed, including altered hepatic energy charge and AMPK activation (98, 122, 123). However, the physiological relevance of these mechanisms has been contested due to the supra-pharmacological (millimolar) concentrations typically used in these studies (48, 50, 55). Additionally, these studies are challenged by conflicting data showing that metformin does not alter hepatic energy charge and does not require AMPK activation to exert its therapeutic effects in vivo (59, 124). In this section, we review the studies related to complex I inhibition by metformin.

Complex I inhibition at millimolar concentrations of metformin is well-established, and metformin is proposed to alter hepatic adenine nucleotide energy charge due to decreased electron transport chain activity (58, 98). Specifically, reduced electron transport chain activity decreases the cellular [ATP]:[ADP] and [ATP]:[AMP] ratios, which may potentially mediate the antidiabetic effects of metformin.

One study reported that increased hepatic AMP concentrations following metformin treatment allosterically inhibits adenylyl cyclase, decreasing intracellular cAMP production and antagonizing hepatic glucagon action (98). However, we and others have not observed an effect of metformin on cAMP levels at clinically relevant concentrations of metformin (50, 59, 125). Moreover, a trial of metformin in individuals with prediabetes showed that metformin did not suppress glucagon-dependent HGP, demonstrating that this mechanism for metformin action does not appear to be relevant to humans (71).

It should be noted that Miller et al propose an additional mechanism, in which AMP directly inhibits gluconeogenesis through allosteric inhibition of fructose 1,6-biphosphatase (98). In support of this mechanism, a recent study reported that expression of a mutant fructose 1,6-bisphosphatase enzyme that is not regulated by AMP abrogated the glucose-lowering effect of metformin in vivo (61). However, the oral dose of metformin (250 mg/kg) used in this study was very high, resulting in plasma concentrations of metformin that were nearly 10-fold higher than those measured in patients with T2D taking metformin (46). Thus, further investigation is needed to determine the clinical significance of this mechanism.

Whether clinically relevant concentrations of metformin alter hepatocellular adenine nucleotide levels is also unresolved. As discussed earlier, one of the main challenges in interpreting these studies as well as other studies that have implicated complex I as the major therapeutic target of metformin is the supra-pharmacological doses of metformin utilized in many studies (57-60, 63, 124, 126-128) (Tables 1, 2). Initial studies indicated that, in vitro, the K_0.5_ for metformin inhibition of complex I in submitochondrial particles was 79 mM (57). Subsequent studies have observed complex I inhibition only in the presence of 2 mM to 10 mM and, similarly, altered hepatic energy charge is not observed at clinically relevant concentrations, which are in the micromolar range (58, 59, 124). Furthermore, studies in lean and rat models of T2D report no change in hepatic [ATP]:[ADP] or [ATP]:[AMP] ratios following acute or chronic metformin treatment (48, 50).

### AMPK activation

In addition to complex I inhibition, one of the most frequently invoked mechanisms of metformin action is AMPK activation due to complex I inhibition. AMPK is a cellular energy sensor that interacts with adenine nucleotides to promote Thr172 phosphorylation by liver kinase B1 (LKB1) or Ca^2+^/calmodulin-dependent protein kinase kinase β (CAMKKβ) (129). AMPK, which forms a heterotrimeric complex, is bound to ATP in the basal state and, as the concentrations of ATP/ADP/AMP change in the cell, ATP is replaced with either ADP or AMP, allosterically activating AMPK. This occurs during times of metabolic stress, such as prolonged starvation and intense exercise (129, 130). Based on the observations that metformin inhibits complex I and reduces the [ATP]:[ADP] and [ATP]:[AMP] ratios, AMPK was a promising candidate to explain metformin’s antidiabetic effects. The proposed beneficial metabolic effects of AMPK activation are two-fold: transcriptional downregulation of gluconeogenic genes reduces HGP, and phosphorylation of acetyl-CoA carboxylase 1 (ACC1) and ACC2, which reduces lipogenesis and promotes hepatic mitochondrial oxidation, respectively (131), resulting in reductions in hepatic diacylglycerol content and improved hepatic insulin sensitivity (122, 123, 132).

Although there is disagreement in the literature regarding hepatic energy charge as a mechanism of metformin action, metformin is shown to activate AMPK-Thr172 phosphorylation independently of changes in adenine nucleotides (48, 124, 125). However, it is unlikely that metformin directly binds to and activates AMPK because metformin has no effect on the activity of purified AMPK (123). In the search for a new mechanism of AMPK activation, LKB1 was identified in the early 2000s as an upstream kinase responsible for phosphorylating and activating AMPK and was implicated as a major target of metformin action (122, 133). Activation of this pathway induces disassembly of the CREB-CBP-CRTC2 complex which transcriptionally regulates gluconeogenic gene expression (91, 92).

In support of this mechanism, liver-specific LKB1 knockout mice presented with hyperglycemia, inactivation of AMPK, and transcriptional upregulation of gluconeogenesis. Importantly, these mice were resistant to metformin’s glucose-lowering effect, suggesting a LKB1-AMPK-CRTC2-dependent metformin mechanism of action (122). However, conflicting data were reported by Foretz et al, demonstrating that LKB1 knockout hepatocytes were surprisingly responsive to metformin therapy, and liver-specific deletion of AMPK was insufficient to suppress metformin action (59). The notable discrepancies between these studies may be due to variation in metformin dosage, route of administration (intraperitoneal versus intragastric), and diet composition (regular chow versus high fat).

ACC1 and ACC2 are major downstream targets of AMPK activation that are involved with the regulation of lipid metabolism. Specifically, ACC1 and ACC2 catalyze the production of malonyl-CoA, a precursor for *de novo* lipogenesis and a regulator of mitochondrial fat oxidation (134-136). Thus, phosphorylation and inhibition of ACC1 and ACC2 by AMPK decreases hepatic lipogenesis and increases hepatic fat oxidation respectively (131), leading to reduced hepatic lipid accumulation and improved insulin sensitivity. In an elegant study using ACC double knock-in (DKI) mice that are insensitive to AMPK inhibition, Fullerton et al showed that AMPK inhibition of ACC is necessary for the therapeutic actions of chronic metformin treatment in mice fed a high-fat diet (132). Obese ACC DKI mice were insensitive to metformin’s chronic effect to decrease plasma glucose and improve hepatic lipid handling, indicating an essential role for ACC1 and ACC2 in this process. Interestingly, the authors show that these mice were in fact responsive to acute metformin treatment. This is consistent with a later study using the same ACC DKI mouse model, which showed no genotype differences in response to acute metformin treatment following high-fat feeding (48), suggesting an ACC-independent mechanism.

If metformin’s mechanism of action is dependent on the LKB1-AMPK-ACC pathway, it would be expected that chronic metformin treatment would alleviate nonalcoholic fatty liver disease (NAFLD). Indeed, multiple groups have shown that both pharmacological inhibition of hepatic ACC and activation of AMPK independently reverse hepatic steatosis and restore hepatic insulin sensitivity in rodents, nonhuman primates, and humans (137-139). In contrast, metformin is unable to reverse hepatic steatosis or improve liver function in the absence of weight loss in nondiabetic patients (140). Furthermore, a randomized, placebo-controlled trial in patients with T2D showed that metformin did not alter hepatic lipid accumulation or fat oxidation, indicating that metformin-induced improvements in lipid metabolism are likely secondary to weight loss and/or improved glycemic control (141). Taken together, these data suggest that hepatic ACC inhibition is not a major target of metformin action in humans.

In summary, there is competing evidence both in support of and in opposition to an AMPK-dependent mechanism of action for therapeutic doses of metformin. AMPK modestly contributes to metformin’s beneficial metabolic effects; however, it is unlikely that AMPK activation is fundamentally required for metformin action to inhibit hepatic gluconeogenesis and reduce rates of HGP in patients with T2D based on the current literature.

### Increased cytosolic redox state

A more recently proposed mechanism of action of metformin is increased cytosolic redox due to inhibition of hepatic GPD2 activity (Fig. 2, bottom panel). In the liver, glycerol is phosphorylated to G3P and converted to DHAP by GPD2. Thus, GPD2 is necessary for glycerol entry into the gluconeogenic pathway. GPD2 is also a redox-dependent enzyme that is a key component of the α-glycerophosphate shuttle, 1 of the 2 major redox shuttles (the second being the malate-aspartate shuttle). These redox shuttles play a key role in the maintenance of cytosolic and mitochondrial redox balance by transferring reducing equivalents between the 2 compartments, leading to alterations in the [NADH]:[NAD^+^] ratio (142-144). Importantly, GPD2 inhibition reduces gluconeogenesis from redox-dependent substrates only (glycerol and lactate), which differentiates this mechanism from what would be expected with complex I inhibition as well as all other proposed mechanisms for metformin action. This substrate selectivity for metformin inhibition of hepatic gluconeogenesis has been demonstrated both in vitro and in vivo (48, 145). Contrary to these findings, Calza et al did not report any changes in gluconeogenesis from lactate in response to metformin in the perfused rat liver (146) and Alshawi et al did not observe significant inhibition of GPD2 by metformin (147). However, in contrast to these 2 studies, several other groups have independently reported an inhibitory effect of metformin and phenformin on GPD2 activity at clinically relevant (μM) concentrations (50, 148-150). Metformin inhibition of GPD2 activity is further supported by studies showing increased hepatocellular G3P and glycerol concentrations following metformin treatment in vitro and in vivo, which is consistent with inhibition of GPD2 activity (50, 151).

GPD2 is widely expressed throughout the body; however, expression levels vary notably between tissues (152-154). A recent report from MacDonald et al questioned the proposed GPD2-dependent mechanism of metformin action in the liver, in part due to the high level of pancreatic GPD2 expression (155). Thus, the authors concluded that because metformin does not inhibit pancreatic GPD2 or block insulin secretion from the β-cell, it is unlikely that GPD2 is metformin’s main target. However, this conclusion fails to consider the tissue distribution of metformin. As discussed earlier, metformin primarily accumulates in the liver, kidney, and small intestine due to the expression profile of the OCT1, OCT3, and MATE1 transporters, which are required for metformin uptake (64, 65, 156, 157). Indeed, metformin treatment was recently shown to alter redox balance in the kidney in addition to the liver (48, 158), consistent with GPD2 inhibition in tissues in which metformin accumulates (159).

It has also been suggested that metformin inhibits the malate-aspartate shuttle; however, this mechanism is challenged by the absence of an effect of metformin on malate dehydrogenase or aspartate aminotransferase (50, 147). Furthermore, although the malate-aspartate shuttle may compensate for long-term changes in redox balance, this cannot negate the impact of GPD2 inhibition on glycerol’s contribution to gluconeogenesis. As described in the preceding sections, glycerol turnover is increased in individuals with T2D due to insulin resistance and inflammation in WAT, which in turn leads to increased contributions of glycerol to gluconeogenesis by a substrate-push mechanism (111, 114, 115). There is also evidence to suggest that metformin can alter redox balance even when rates of the glycerophosphate shuttle do not exceed that of the malate-aspartate shuttle (160-162).

Data obtained from GPD2 knockout mice and patients with GPD2 mutations or deficiency have provided insight to the metabolic consequences of GPD2 inhibition (50, 163-165). Most striking is the observation that GPD2 knockout mice are protected from diet-induced hyperglycemia independent of glucose-stimulated insulin secretion (165). Perturbation of the glycerophosphate shuttle also inhibits gluconeogenesis from glycerol, leading to impaired lipid and amino acid metabolism in mice. The clinical relevance of these changes is demonstrated by the association between low GPD2 expression and hepatic steatosis in patients with and without NAFLD, potentially indicating reduced gluconeogenesis from glycerol (164).

In view of these studies, there is strong support for GPD2 inhibition/cytosolic redox modulation as a mediator of metformin’s therapeutic effect. In the following sections, we summarize the evidence for a direct versus indirect mechanism of inhibition, in addition to the redox-dependency of this mechanism.

### Direct versus indirect inhibition of GPD2 by metformin

Metformin is shown to decrease hepatic gluconeogenesis from redox-dependent substrates through inhibition of GPD2 activity; however, it is unclear whether metformin directly or indirectly inhibits GPD2 (48, 50). Importantly, metformin inhibition of GPD2 exhibits noncompetitive kinetics with a K_i_ of ~50µM, which is in stark contrast with the millimolar concentrations required to inhibit complex I activity. Supporting a direct interaction between metformin and GPD2, independent studies using intact mitochondria, mitochondrial lysates, and isolated enzyme assays with immunoprecipitated GPD2, biguanides are shown to inhibit GPD2 activity in vitro (50, 148, 150), although other groups have not observed an inhibitory effect (147).

Several alternative hypotheses in support of an indirect mechanism of GPD2 inhibition have also been postulated. It is likely that metformin has many additional effects and may in fact alter the activity of several complexes of the electron transport chain, which in turn indirectly leads to inhibition of GPD2. Metformin and other guanides have consistently been shown to bind metal ions, such as copper and iron, possibly by acting as a Schiff base, which is consistent with reports that metformin interacts with heme or cytochrome c directly (166-168). Indeed, downstream inhibition of the electron transport chain is shown to backlog the entire electron transport chain, providing a potential link between metformin interaction with downstream complexes and indirect inhibition of GPD2 (169, 170). GPD2 is also calcium-dependent and is therefore sensitive to changes in local calcium concentrations; however, there is little evidence to support a role for metformin in calcium homeostasis (171). Further investigation is necessary to determine whether metformin and other guanides interact with copper and iron contained in the electron transport chain, which in turn results in inhibition of GPD2 by an indirect mechanism leading to an increase in the cytosolic redox state. Metformin-metal interactions might also explain the pleotropic effects of metformin on mitochondrial function and other metabolic processes.

## Summary

The observation that metformin selectively inhibits gluconeogenesis from redox-dependent substrates (lactate and glycerol), but not redox-independent substrates (pyruvate, DHAP, alanine) strongly indicates a redox-dependent mechanism of action (37, 145, 172). Several groups have shown that biguanide treatment increases the cytosolic redox state in a variety of tissues, consistent with inhibition of the glycerophosphate shuttle (48, 116, 148, 151, 158, 159, 173, 174). Metformin-induced increases in the cytosolic redox state will reduce lactate conversion to pyruvate due to the dependency of lactate dehydrogenase activity on the cytosolic redox state and it will reduce glycerol conversion to glucose due to inhibition of GPD2 activity.

Isotopically labeled tracer studies have independently confirmed that metformin reduces gluconeogenesis from lactate and glycerol, while gluconeogenesis from alanine remains unaltered (37, 48, 145). Moreover, restoring cytosolic redox to normal levels was sufficient to abrogate metformin’s glucose-lowering effect (48, 145, 172, 175). Importantly this substrate-selective inhibition of gluconeogenesis cannot be explained by any of the previously described proposed mechanisms of metformin action, including complex I inhibition, AMPK activation, fructose 1,6-bisphosphatase inhibition, or CREB inhibition. Furthermore, this redox/substrate–dependent mechanism may account for the remarkable safety profile of metformin. In comparison with other pharmacologic interventions for T2D, metformin treatment rarely causes hypoglycemia (176). This is consistent with metformin’s substrate-specific inhibition of gluconeogenesis from lactate and glycerol and lack of inhibition of gluconeogenesis from amino acids.

When considering the numerous reports demonstrating metformin’s pleiotropic effects, it is likely that metformin has several molecular targets. Yet, this remains an area of active investigation. Here, we have highlighted several characteristics of metformin action that are consistent with a redox-dependent mechanism of action on hepatic gluconeogenesis at therapeutically relevant (50-100 μM) concentrations. Furthermore, we described key features of metformin treatment that are inconsistent with clinically meaningful complex I inhibition, including the millimolar concentrations required to observe complex I inhibition, and the substrate selectivity of metformin inhibition of gluconeogenesis. Many of the postulated mechanisms of metformin action, including AMPK activation and altered energy charge, are dependent on significant complex I inhibition. Therefore, although these pathways may play a role in the pleiotropic effects of metformin, they are likely dispensable for the glucose-lowering effect of metformin in patients with poorly controlled T2D. In recent years, the utility of metformin has been expanded beyond the first-line treatment for T2D, and it has the potential to enter the realms of aging, cancer, and cardiovascular disease. Thus, the quest to identify a clear mechanism of metformin action is pertinent to the development of new therapeutic strategies and alleviating these chronic illnesses.

## Data Availability

Data sharing is not applicable to this article as no datasets were generated or analyzed during the current study.

## Abbreviations

ACC: acetyl-CoA carboxylase
AMPK: adenosine monophosphate–activated protein kinase
CBP: CREB-binding protein
CREB: cAMP-responsive element-binding protein 1
CRTC2: CREB-regulated transcription co-activator 2
DHAP: dihydroxyacetone phosphate
DKI: double knock-in
FOXO: Forkhead box O
G3P: glycerol-3-phosphate
GDF15: growth differentiation factor 15
GLP-1: glucagon-like peptide-1
GPD: glycerol-3-phosphate dehydrogenase
HGP: hepatic glucose production
LKB1: liver kinase B1
NAFLD: nonalcoholic fatty liver disease
NEFA: nonesterified fatty acid
OCT1: organic cation transporter 1
PET: positron emission tomography
T2D: type 2 diabetes
UKPDS: UK Prospective Diabetes Study
WAT: white adipose tissue

## Additional Information

***Disclosure Summary:*** G.I.S. serves on the scientific advisory boards for Merck, AstraZeneca, iMBP, and Janssen Research and Development and receives investigator-initiated support from Gilead Sciences, Merck, and AstraZeneca. He is also a Scientific Co-Founder of TLC. Inc.
